# Disease Evolution Monitored by Serial Cerebrospinal Fluid Liquid Biopsies in Two Cases of Recurrent Medulloblastoma

**DOI:** 10.3390/ijms25094882

**Published:** 2024-04-30

**Authors:** Katrina O’Halloran, Ashley Margol, Tom B. Davidson, Dolores Estrine, Benita Tamrazi, Jennifer A. Cotter, Jianling Ji, Jaclyn A. Biegel

**Affiliations:** 1 Cancer and Blood Disease Institute, Children’s Hospital Los Angeles, Los Angeles, CA 90027, USA; kohalloran@chla.usc.edu (K.O.); amargol@chla.usc.edu (A.M.); tdavidson@chla.usc.edu (T.B.D.); 2 Keck School of Medicine, University of Southern California, Los Angeles, CA 90033, USA; btamrazi@chla.usc.edu (B.T.); jcotter@chla.usc.edu (J.A.C.); jji@chla.usc.edu (J.J.); 3 Department of Pathology and Laboratory Medicine, Children’s Hospital Los Angeles, Los Angeles, CA 90027, USA; destrine@chla.usc.edu; 4 Department of Radiology, Children’s Hospital Los Angeles, Los Angeles, CA 90027, USA

**Keywords:** medulloblastoma, relapse, liquid biopsy, cerebrospinal fluid

## Abstract

Medulloblastoma is the most common malignant brain tumor in childhood. Initial treatment generally includes surgery, irradiation, and chemotherapy. Approximately 20–30% of patients will experience a recurrence, which portends a very poor prognosis. The current standard of care for evaluation for relapse includes radiographic surveillance with magnetic resonance imaging at regular intervals. The presence of circulating tumor DNA in the cerebrospinal fluid has been demonstrated to be a predictor of a higher risk of progression in a research setting for patients with medulloblastoma treated on a prospective single institution clinical trial. We have previously published and clinically validated a liquid-biopsy-based genetic assay utilizing low-pass whole genome sequencing to detect copy number alterations in circulating tumor DNA. Here, we present two teenage patients with posterior fossa medulloblastoma with recurrent disease who have been monitored with serial liquid biopsies showing tumor evolution over time, demonstrating the clinical utility of these approaches.

## 1. Introduction

Medulloblastoma is the most common malignant central nervous system (CNS) tumor in children [1,2]. Upfront treatment of medulloblastoma generally includes maximal safe surgical resection followed by craniospinal irradiation (CSI) and chemotherapy [3,4]. Unfortunately, medulloblastoma recurs in approximately 20–30% of patients and survival following recurrence is poor [5,6].

Advances in tumor molecular profiling have identified four distinct molecular groups of medulloblastoma: wingless (WNT), sonic hedgehog (SHH), Group 3, and Group 4 [7,8]. A variety of clinical assays have been implemented for medulloblastoma grouping. The molecular subgroup is predictive of survival and there are known subgroup-specific therapeutic vulnerabilities [9,10,11].

Cerebrospinal fluid (CSF) has been shown by our group and others to contain circulating tumor DNA (ctDNA) based on the presence of copy number aberrations (CNAs) reflective of the molecular cytogenetic signature of the primary tumor [12,13,14]. Detection of ctDNA at the end of medulloblastoma therapy has been shown to predict recurrence when serially banked CSF samples from children enrolled on a clinical trial were evaluated for CNAs [15]. However, how to optimally incorporate liquid biopsy results prospectively on an individual basis to modify clinical treatment remains an outstanding question.

The Center for Personalized Medicine at Children’s Hospital Los Angeles (CHLA) has developed a platform for CSF and plasma ctDNA assessment in children with CNS and solid tumors, respectively, which utilizes low-pass whole genome sequencing (LP-WGS) for the detection of CNAs, as previously described [12,13]. The LP-WGS portion of this platform is now validated in a CAP/CLIA-certified environment and is available at CHLA as an orderable test with results returned to the clinician as a formalized pathology report within the electronic health record. The test and results are being incorporated into clinical practice and may be used in conjunction with the patient’s overall clinical picture to guide and optimize care at the clinician’s discretion. 

## 2. Case Presentation

### 2.1. Case 1

**Figure 1:**
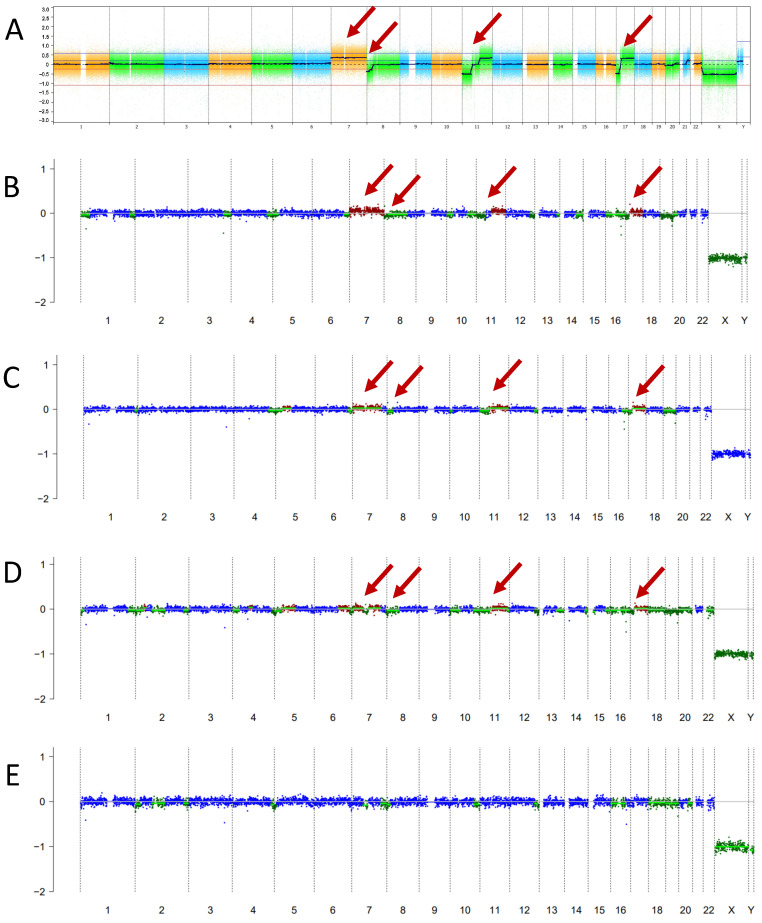
Primary tumor profiling and subsequent serial liquid biopsies for Case 1. (**A**) Primary tumor chromosomal microarray. (**B**) Initial liquid biopsy at EOT. (**C**–**E**) Serial liquid biopsy samples over time at 3 months, 6 months, and 9 months from initial liquid biopsy sampling, showing the resolution of CNAs. Red arrows point to the gain of chromosome 7, loss of 8p, loss of 11p and gain of 11q, and isodicentric 17q.

Case 1 is a now 13-year-old male of Asian descent with refractory Group 4 medulloblastoma. He initially presented at 11 years of age with several months of headaches and nausea/vomiting. On magnetic resonance imaging (MRI), he was found to have a posterior fossa mass centered in the fourth ventricle, with disseminated subependymal and leptomeningeal disease; MRI of the spine was negative for disease. He underwent sub-total resection which showed medulloblastoma with classic histology. CSF cytology was negative for malignant cells. Chromosomal microarray analysis showed a gain of chromosome 7, loss of 8p, loss of 11p and gain of 11q, gain of 14q22.3, and an isodicentric chromosome 17q (Figure 1A). There was no evidence of *MYC* or *MYCN* amplification. Methylation profiling was consistent with Group 4 medulloblastoma. Given his high-risk clinical features (residual and metastatic disease), he was treated as per the COG study ACNS0332 (NCT00392327) with 36 Gy craniospinal irradiation plus a 20 Gy boost, followed by 6 cycles of cisplatin and cyclophosphamide-based maintenance chemotherapy.

**Figure 2:**
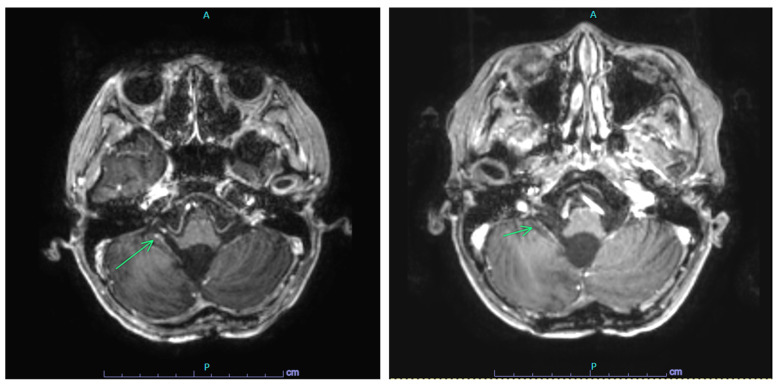
MRI findings in Case 1 EOT scan (**left**) with a nodular focus (arrow) and on-therapy subsequent scan (**right**) with resolution of nodular focus (arrow) and no evidence of disease. A = anterior; P = posterior.

Due to significant toxicities, his maintenance chemotherapy required substantial modifications. His cisplatin was dose-reduced by 25% in Cycle 2 and omitted in Cycles 3–6 due to ototoxicity. His cyclophosphamide was dose-reduced by 25% in Cycles 4 and 5 due to myelosuppression. His end of therapy (EOT) MRI showed a stable size of the fourth ventricular outflow tract nodular focus consistent with “treated disease”, slightly decreased in size from the immediate prior MRI with no new reduced diffusion or abnormal enhancement (Figure 2). His EOT lumbar puncture cytology was negative for malignant cells; however, his liquid biopsy was positive (Figure 1B).

Given his high-risk disease, dose reductions due to toxicities, and persistent nodularity on MRI in the setting of positive CSF liquid biopsy, the decision was made to continue treatment with metronomic anti-angiogenic therapy, with intrathecal chemotherapy via Ommaya reservoir.

Given that he was undergoing serial CSF collections for the administration of intrathecal therapy and assessment of cytology as part of ongoing therapy, he underwent serial CSF liquid biopsies at 3 months, 6 months, and 9 months from initial liquid biopsy sampling (Figure 1C–E, respectively). The nodular focus resolved on subsequent MRI and he remains with no evidence of disease (NED). CSF cytology has also remained negative. Over time, the detection of ctDNAs consistent with medulloblastoma has decreased and eventually resolved, with his most recent liquid biopsy at 9 months negative for ctDNA (Figure 1E). Given his excellent response, he continues on metronomic antiangiogenic therapy and is currently receiving his 11th cycle (total 16 months of therapy thus far). The plan is for ongoing serial monitoring via Ommaya reservoir taps as required as part of his therapy. He has been able to return to school with an excellent quality of life. 

### 2.2. Case 2

Case 2 is a now 18-year-old female of Asian descent with a history of multiply recurrent Group 4 medulloblastoma. She was originally diagnosed at 9 years of age at an outside institution. She underwent gross total resection which showed medulloblastoma with classic histology and had M0 disease at initial staging workup. Primary tumor molecular analysis showed a focal gain of 5q23.2 containing the *SNCAIP* gene, loss of interstitial 4q, and isochromosome 17q, most consistent with Group 4 medulloblastoma. Given her average risk clinical features (gross total resection and no metastatic disease), she was treated as per the COG study ACNS0331 (NCT00085735) with a 23.4 Gy CSI plus boost followed by 9 cycles of maintenance chemotherapy. After first recurrence to the cervical spine at nearly 3 years off therapy, she underwent subtotal resection followed by focal radiation therapy to 30 Gy. She subsequently received one year of therapy with temozolomide/bevacizumab/irinotecan. At further progression in the cervical spine with fourth ventricular leptomeningeal disease 6 months later, she was treated on a phase I clinical trial and at the time of further progression after 5 months transferred care to CHLA. Re-induction with cyclophosphamide, irinotecan, temozolomide, and etoposide per institutional protocol was attempted; however, there was a minimal response. She was therefore then started on metronomic antiangiogenic therapy with intrathecal therapy and serial CSF evaluations. 

**Figure 3:**
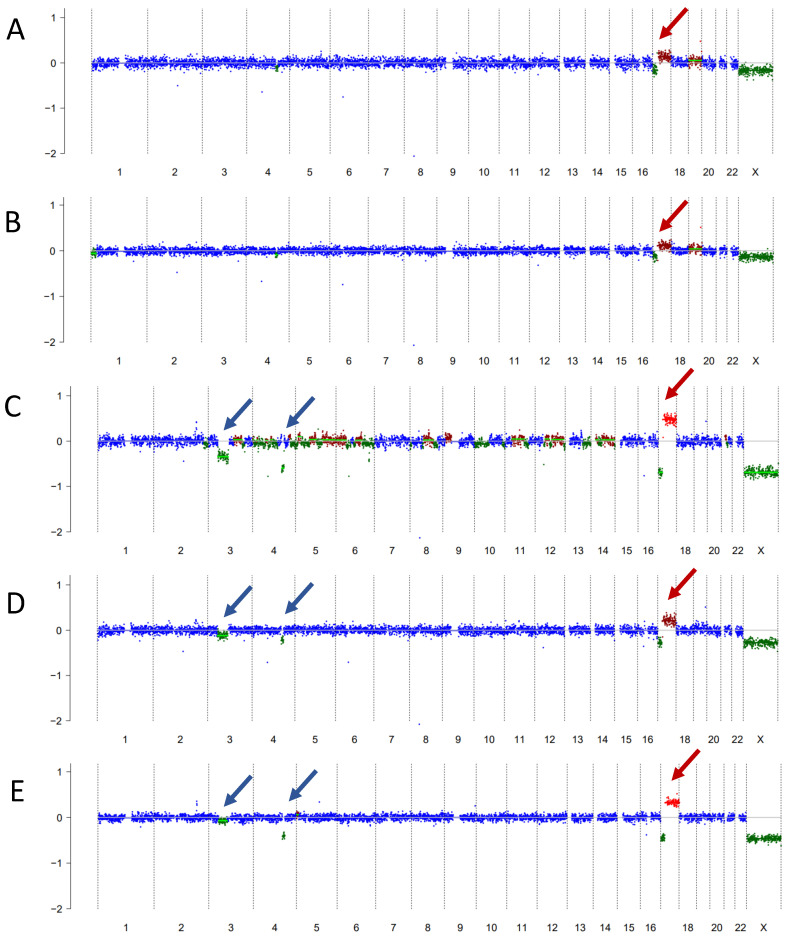
(**A**–**E**) Serial liquid biopsies for Case 2. (**A**) is from after Cycle 6 of metronomic therapy; (**B**–**E**) are subsequent samples at 3 months, 6 months, 9 months, and 12 months from initial liquid biopsy, showing tumor evolution over time. Red arrows show the consistent isochromosome 17q. New deletions in chromosomes 3 and 4 are indicated by blue arrows.

**Figure 4:**
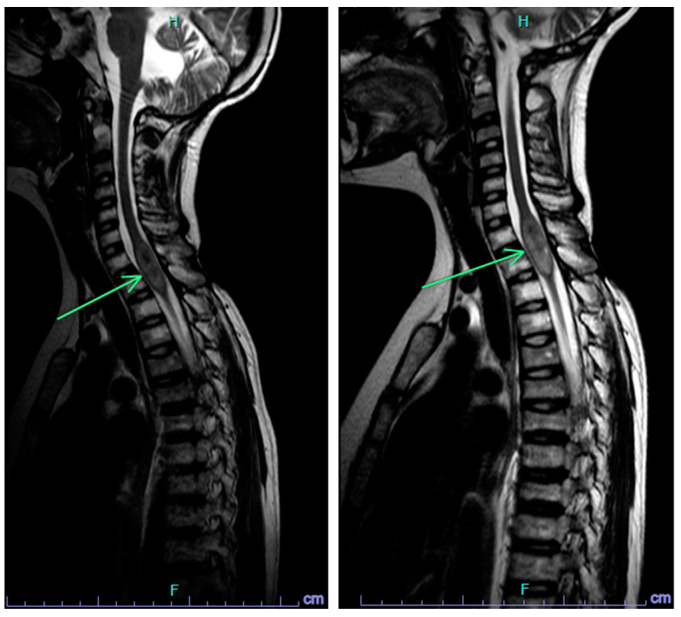
Case 2 immediate pre-progression scan (**left**) showing a stable cervical spinal lesion, and follow-up scan (**right**) with progression of the cervical spinal lesion (arrow).

The liquid biopsy test became available at CHLA after Cycle 6 of metronomic therapy and was positive for ctDNA (Figure 3A). The first CSF sample demonstrated an isochromosome 17q, which was also present in all subsequent serial liquid biopsy samples at 3 months, 6 months, 9 months, and 12 months (Figure 3B–E). She remains on therapy and is currently receiving her 16th cycle (total 21 months of therapy thus far). Her serial liquid biopsies over time demonstrated additional CNAs consistent with clonal evolution concerning for tumor change. Her MRI scans initially remained stable, except following the most recent liquid biopsy, when a slight increase in the size of the cervical spinal lesion was again noted (Figure 4). CSF cytology has remained negative throughout. The information from her liquid biopsies which pre-dated the MRI findings has allowed the clinical team and family time for ongoing discussion and planning for next steps in therapy, when the need arises.

## 3. Discussion

The two cases demonstrate that serial liquid biopsy sampling is feasible and informative. Our CSF platform for the detection of ctDNA by LP-WGS for CNA detection is optimized for 3–5 mL of CSF and 5 ng of input DNA; however, smaller volumes and yields may yield successful results [12]. Of note, CSF collection is feasible particularly with protocols such as metronomic anti-angiogenic therapies, which include a component of intrathecal therapy and ready ongoing access to CSF [16,17]. However, given the clinical utility and the minimal risk associated with lumbar puncture, we would advocate for lumbar puncture for CSF sampling in patients without Ommaya reservoirs. Our decision to monitor the above cases every three months was made in order to coincide with MRI evaluations as an additional reference point and as the standard of care in disease evaluation. 

As shown in Case 1, the results of the liquid biopsy are highly concordant with the molecular characteristics of the primary tumor tissue. We (and others) have shown this to be true in the setting of medulloblastoma and in other embryonal and non-embryonal pediatric CNS tumor types [12,13,18,19]. We have also shown, as in Case 2, the utility of liquid biopsy for the detection of tumor evolution, based on the presence of additional CNAs noted over time, and in her case pre-dating radiographic tumor changes. 

The identification of new molecular alterations is important for prognosis and treatment decision making; however, performing serial tumor tissue biopsies is often not feasible, particularly from morbidity, quality of life, and also healthcare cost perspectives, making serial liquid biopsy for evaluation of tumor evolution an appealing alternative. Other groups have also shown evidence for tumor evolution in ctDNA, including in the setting of gliomas [19]. The utility of liquid biopsy in monitoring the response to therapy has also been shown, as in the case of diffuse midline glioma treated with radiation therapy and also in the context of treatment with novel agents including ONC201 [20,21].

The recent publication from Liu et al. highlights the prognostic value of liquid biopsy in so-called minimal residual disease detection in medulloblastoma and showed that patients with detectable disease at the end of therapy had a higher risk of relapse [15]. These results highlight how liquid biopsy may help to identify patients at increased risk. However, how to use liquid biopsy in real time in clinical decision-making still requires further investigation. Case 1 was considered to have refractory disease requiring additional therapy based on the clinical picture of insufficient therapy, concerning MRI findings, and a positive liquid biopsy. Case 2 highlights the use of liquid biopsy in monitoring tumor evolution and illustrates how liquid biopsy may pre-date the standard imaging findings of recurrence.

Liquid biopsy-based genomic analysis is a novel, clinically validated platform, although further work is needed to best understand the optimal use in clinical care and treatment decision-making. Future directions include building upon proof-of-principle pilot studies with the evaluation of serial sampling for a better understanding of the natural history, and with prognostic correlates, on a larger scale and in additional tumor types. Implementation into prospective clinical trials should be considered in medulloblastoma and other CNS tumor types.

## 4. Materials and Methods

Details regarding the development of the liquid biopsy platform at Children’s Hospital Los Angeles have been previously described in prior publications [12,13]. Signed informed consent for inclusion in this case report was obtained in both cases.

## 5. Conclusions

In conclusion, CSF liquid biopsy-based genomic profiling may facilitate the monitoring of patients with CNS tumors as shown in these two cases of recurrent medulloblastoma. Incorporation into large-scale clinical trials is recommended in order to determine more specific guidelines for routine clinical care.

## Data Availability

Not applicable.
